# Effect of poly-hexamethylene biguanide hydrochloride (PHMB) treated non-sterile medical gloves upon the transmission of *Streptococcus pyogenes*, carbapenem-resistant *E. coli*, MRSA and *Klebsiella pneumoniae* from contact surfaces

**DOI:** 10.1186/s12879-017-2661-9

**Published:** 2017-08-17

**Authors:** S. Ali, A.P.R. Wilson

**Affiliations:** 1 Clinical Microbiology and Virology, University College London Hospitals NHS Foundation Trust, London, UK; 2 UCLH Environmental Research Laboratory, University College Hospital, EGA Wing, Level -2, 235 Euston Road, London, NW1 2BU UK

**Keywords:** PHMB, Gloves, Hand hygiene

## Abstract

**Background:**

Reduction of accidental contamination of the near-patient environment has potential to reduce acquisition of healthcare-associated infection(s). Although medical gloves should be removed when soiled or touching the environment, compliance is variable. The use of antimicrobial-impregnated medical gloves could reduce the horizontal-transfer of bacterial contamination between surfaces.

**Aim:**

Determine the activity of antimicrobial-impregnated gloves against common hospital pathogens: *Streptococcus pyogenes*, carbapenem-resistant *E.coli* (CREC), MRSA and ESBL-producing *Klebsiella pneumoniae*.

**Methods:**

Fingerpads (~1cm^2^) of PHMB-treated and untreated gloves were inoculated with 10 μL (~10^4^ colony-forming-units [cfu]) of test-bacteria prepared in heavy-soiling (0.5%BSA), blood or distilled-water (no-soiling) and sampled after 0.25, 1, 10 or 15 min contact-time.

Donor surfaces (~1cm^2^ computer-keys) contaminated with wet/dry inoculum were touched with the fingerpad of treated/untreated gloves and subsequently pressed onto recipient (uncontaminated) computer-keys.

**Results:**

Approximately 4.50log_10_cfu of all bacteria persisted after 15 min on untreated gloves regardless of soil-type. In the absence of soiling, PHMB-treated gloves reduced surface-contamination by ~4.5log_10_cfu (>99.99%) within 10 min of contact-time but only ~2.5log_10_ (>99.9%) and ~1.0log_10_ reduction respectively when heavy-soiling or blood was present.

Gloves became highly-contaminated (~4.52log_10_–4.91log_10_cfu) when handling recently-contaminated computer-keys. Untreated gloves contaminated “recipient” surfaces (~4.5log_10_cfu) while PHMB-treated gloves transferred fewer bacteria (2.4–3.6log_10_cfu). When surface contamination was dry, PHMB gloves transferred fewer bacteria (0.3–0.6log_10_cfu) to “recipient” surfaces than untreated gloves (1.0–1.9log_10_; *P* < 0.05).

**Conclusions:**

Antimicrobial-impregnated gloves may be useful in preventing dissemination of organisms in the near-patient environment during routine care. However they are not a substitute for appropriate hand-hygiene procedures.

## Introduction

In European hospitals, 5.7% of patients have a healthcare associated infection at any one time [1]. These infections form a quarter of all patient-adverse events and cost 7 billion Euros per year [2]. The emergence of multidrug resistant Gram negative pathogens has made efforts for the reduction of healthcare-associated infection a priority. Staff hand hygiene is thought to be the most effective method to prevent transmission of these pathogens. In critical care the most frequently touched surfaces are the bedside computer and equipment trolley [3]. Hand hygiene compliance between computer keyboards and surfaces within the vicinity and the patient is poor. Adoption of electronic patient records with hand held tablet computers on patient wards increases risk of transmission especially as bacteria and spores may survive there for several weeks. Although hospital infection prevention policies require removal of gloves and hand hygiene between patient contact and touching nearby surfaces, this may not be performed, particularly in the emergency situation. Soiling of gloves may not be visible to the wearer yet harbour pathogenic organisms. Soiled/contaminated gloves should be removed between activities but this may not be immediate. Once organisms are transferred to any nearby surface there is a potential for further dissemination [4, 5].

An antimicrobial additive to the glove material could reduce the risks associated with accidental contact with surfaces. The antimicrobial-efficacy of poly-[hexa-methylene]-biguanide-hydrochloride (PHMB)-treated non sterile medical gloves was tested against a range of bacteria associated with healthcare infections. The effect on transfer of organisms to and from computer-keyboard surfaces under various levels of soiling is assessed.

## Methods

### Preparation and validity-testing of the glove material

#### Preparation of test organisms

Clinical isolates of carbapenem-resistant *E.coli* (CREC), meticillin-resistant *Staphylococcus aureus* (MRSA; EMRSA-15 variant B1), an extended-spectrum beta-lactamase (ESBL)-producing *Klebsiella pneumoniae* and a type-culture of *Streptococcus pyogenes* ATCC 19615 were tested.

Overnight culture of the test bacteria (10 μL) was transferred to sterile nutrient broth (10 mL; Oxoid, UK), mixed thoroughly and incubated aerobically at 37 °C for 18 h. Broth cultures were centrifuged at 3000 rpm (1500 × g; Jouan CR3i centrifuge: Thermo, UK) for 10 min and the remaining pellet re-suspended in 10 mL of 0.5% *w*/*v* Bovine Serum Albumin (BSA) (Sigma-Aldrich, UK), sterile horse blood (Oxoid, UK), or no soil (sterile distilled water).

#### Preparation of neutralising solution

Where appropriate, antimicrobial activity was inactivated with a neutralising solution comprising: 3% (*w*/*v*) Tween 80, 0.3% (*w*/*v*) Lecithin, 1.0% (*w*/*v*) Sodium thiosulfate, 1.5% (*w*/*v*) K_2_HPO_4_, KH_2_PO_4_ 0.05% (*w*/*v*), 1% (*w*/*v*) Poly-[sodium-4-styrenesulfonate], 0.1% (*v*/v) Triton® ×100 (Sigma-Aldrich, UK) and prepared in Phosphate-buffered saline (PBS) solution (Oxoid, UK). Solutions were sterilised by autoclaving (121 °C for 15 min) and refrigerated (2–5 °C) until required.

#### Validation of neutraliser efficacy and toxicity

The capacity of the neutraliser to quench the antimicrobial activity of PHMB was confirmed and any toxicity against the test organisms assessed as previously described [6]. An excised square (1 cm x1cm) from an un-used PHMB-treated nitrile glove was immersed in 1 mL sterile de-ionised water and incubated at room temperature for 1 h to allow PHMB to leach into the solution (PHMB leachate). A sterile test tube was dosed with: (A) 1 mL Neutraliser +1 mL PHMB leachate + excised test-glove, (B) 1 mL Neutraliser A + 1 mL Sterile distilled water or (C) 2 mL distilled water (control) and incubated at room temperature for 1 min to allow neutralisation of the PHMB-leachate solution. This was inoculated with 1 mL (10^3^ colony-forming units [cfu]) of *S. pyogenes*, CREC, MRSA or *K. pneumoniae* suspension prepared in sterile distilled water.

The resulting suspensions were incubated a further 10 min at room temperature before spread-plating 0.1 mL onto either Staph/Strep agar (*S. pyogenes* only) or Columbia blood agar plates (CREC, MRSA or *K. pneumoniae*). Plates were incubated aerobically at 37 °C for 48 h.

Neutraliser efficacy (NE) and neutraliser toxicity (NT) were calculated as:

$$ NE={M}_A/{M}_B, and $$

$$ NT={M}_B/{M}_{C,} where $$

M_A_, M_B_ and M_C_ represent the mean number of spores recovered from preparations (A), (B) and control populations (C) respectively. Results were based on 10 replicate test samples.

Using documented criteria^6^, the neutraliser efficacy and toxicity ratios were calculated as being ≥0.75 for all test-preparations (results not shown) demonstrating that the neutraliser was effective against PHMB solution and non-toxic to the test organisms.

### Testing of the glove material

#### Effect of contact time on bacterial inoculum

The finger pad of the index finger of PHMB-treated and untreated (control) gloves was marked with a 1 cm × 1 cm test-area and the area rinsed 3 times with sterile distilled water followed by immersion in 70% ethanol solution before allowing to air-dry. Marked-areas were inoculated with 10uL (~10^4^cfu) of the test organism (*S. pyogenes*, CREC, MRSA or *K. pneumoniae*) suspended in a soil solution (0.5% BSA or sterile horse blood) or in sterile distilled water (DW; control) and incubated at room temperature.

After the appropriate contact time (0.25, 1, 10 or 15 min) at room temperature, test-areas on the finger pad of gloves were excised, transferred to 2 mL neutralising solution and homogenised at high speed for 30 s by vortexing. Assays were incubated in neutraliser for 10 min at room temperature. Serial dilutions of suspensions were performed prior to spread-plating 0.1 mL aliquots either Staph/Strep selective agar (*S. pyogenes* only; Oxoid, UK) or Columbia blood agar plates (CREC, MRSA or *K. pneumoniae*). Plates were incubated aerobically at 37 °C for 48 h prior to reading.

#### Transfer from gloves to recipient-surface

A standard computer keyboard key was prepared as an example recipient-surface. Keys were scrubbed manually using non-chlorinated detergent then rinsed three times using sterile distilled water followed by immersing in 70% ethanol solution and air-dried prior to use.

As previously described (section 2.1), test-areas on treated and untreated (control) gloves were inoculated with 10 μL (~10^4^ cfu) of the test organism prepared in a soil or distilled water (no soil). The inoculated gloves were donned by the investigator either immediately (wet inoculum) or after allowing the inoculum to dry (15 min incubation at room temperature) and the seeded finger-pad pressed onto an un-inoculated recipient surface (computer key) for one second.

The recipient-surface was swabbed immediately with a sterile cotton-tipped swab (pre-moistened in neutralising solution) and the swab transferred to a universal tube containing 2 mL neutralising solution. Swab suspensions were homogenised by vortexing at high speed for 30 s and incubated a further 10 min at room temperature. Aliquots (0.5 mL) of the neat suspension and 0.1 mL from 1/10 and 1/100 serial dilutions were surface-plated onto the appropriate agar and incubated aerobically at 37 °C for 48 h prior to reading.

#### Transfer from donor-surfaces (computer-key) to gloves and computer key

A test-area was marked on a standard computer keyboard key (donor-surface) and a second key (recipient-surface) with a 1 cm × 1 cm square and decontaminated as described previously. Donor-surfaces were seeded with 10 μL (~10^4^ cfu) test organism ±soil-challenge/control soil. An unused treated or untreated glove (control) was donned by the investigator and the finger-pad of the index-finger pressed onto a wet or dry inoculum on the seeded donor-surface for one second. Immediately, the same contaminated finger-pad was pressed onto a second uncontaminated key (recipient-surface).

Donor and recipient-surfaces were swabbed immediately with a sterile cotton-tipped swab (pre-moistened in neutralising solution) and the swab transferred to a universal tube containing 2 mL neutralising solution. Similarly, the finger-pad of each contaminated glove was excised and transferred to a universal tube containing 2 ml neutralising solution. Solutions were vortexed at high speed for 30 s and the resulting suspension incubated in the neutralising solution for 10 min at room temperature. Aliquots (0.5 ml and 0.1 ml) of neat and serial dilutions were plated for incubation as above.

## Results

### Effect of contact time on bacterial inoculum

Test gloves were inoculated with 2.8 × 10^4^- 6.1 × 10^4^ (i.e. 4.45 log_10_–4.78 log_10_) cfu bacteria. Approximately 4.50 log_10_ cfu bacteria could be recovered from both PHMB-treated and untreated gloves when sampled immediately (Table 1).

**Table 1:** Persistence of [Median Log_10_ CFU (Inter-Quartile range)] contaminating bacteria on PHMB-treated or untreated (control) gloves under increasing contact time and soil challenge, *n* = 10

	S. pyogenes		Carbapenem-resistant *E. coli*		MRSA		*K. pneumoniae*	
Contact time (min.)	Un-treated (control) glove	PHMB-treated glove	Un-treated (control) glove	PHMB-treated glove	Un-treated (control) glove	PHMB-treated glove	Un-treated (control) glove	PHMB-treated glove
0.25	4.52 (4.49–4.59)	4.41 (4.35–4.53)	4.26 (4.19–4.33)	4.31 (4.19–4.38)	4.49 (4.34–4.56)	4.33 (4.26–4.58)	4.71 (4.55–4.80)	4.65 (4.53–4.77)
1	4.35 (4.21–4.490)	1.95 (1.84–2.12)	4.66 (4.61–4.68)	2.29 (2.14–2.38)	4.47 (4.34–4.58)	3.49 (3.45–3.57)	4.46 (4.30–4.65)	3.43 (3.26–3.52)
10	3.83 (3.79–3.90)	<0.301	4.33 (4.21–4.35)	<0.301	4.51 (4.38–4.62)	<0.301	4.71 (4.55–4.77)	1.84 (1.73–1.99)
15	2.00 (2.00–2.00)	<0.301	4.28 (4.03–4.36)	<0.301	4.45 (4.34–4.55)	<0.301	4.46 (4.37–4.58)	1.80 (1.38–1.97)
0.25	4.42 (4.33–4.51)	4.44 (4.28–4.58)	4.31 (4.19–4.44)	4.42 (4.29–4.51)	4.37 (4.29–4.58)	4.47 (4.33–4.54)	4.65 (4.58–4.79)	4.65 (4.48–4.76)
1	4.61 (4.49–4.72)	3.98 (3.81–4.07)	4.72 (4.58–4.75)	3.46 (3.37–3.60)	4.54 (4.41–4.69)	3.61 (3.36–3.65)	4.68 (4.48–4.80)	3.47 (3.33–3.57)
10	4.80 (4.67–4.84)	2.41 (2.29–2.45)	4.30 (4.22–4.350	3.23 (3.05–3.30)	4.39 (4.25–4.60)	1.86 (1.76–2.03)	4.56 (4.49–4.63)	2.06 (1.95–2.10)
15	4.70 (4.54–4.77)	1.76 (1.57–2.04)	4.35 (4.24–4.45)	2.46 (2.33–2.56)	4.53 (4.49–4.67)	2.11 (1.92–2.19)	4.58 (4.60–4.70)	1.84 (1.56–1.89)
0.25	4.52 (4.43–4.60)	4.57 (4.48–4.62)	4.39 (4.31–4.42)	4.38 (4.25–4.46)	4.49 (4.35–4.60)	4.65 (4.46–4.69)	4.67 (4.56–4.78)	4.74 (4.58–4.81)
1	4.58 (4.49–4.71)	3.97 (3.85–4.08)	4.69 (4.65–4.72)	3.87 (3.82–3.93)	4.58 (4.46–4.67)	3.60 (3.53–3.64)	4.75 (3.26–3.52)	3.64 (3.53–3.73)
10	4.63 (4.56–4.69)	3.89 (3.70–3.95)	4.29 (4.11–4.42)	3.29 (3.19–3.32)	4.58 (4.45–4.66)	3.42 (3.32–3.47)	4.64 (4.54–4.70)	3.46 (3.25–3.54)
15	4.74 (4.69–4.82)	3.87 (3.80–3.93)	4.26 (4.18–4.35)	3.16 (2.33–2.56)	4.49 (4.45–4.58)	3.44 (3.25–3.55)	4.67 (4.60–4.71)	3.26 (3.10–3.32)

Note: Numbers below the detection limit (2 CFU) are expressed as <0.301 Log_10_ numbers

Untreated gloves remained contaminated with high numbers (4.50 log_10_ cfu) for up to 15 min with the test organisms regardless of the soil type (distilled water, 0.5% BSA, Blood). The only exception was *S. pyogenes,* where the absence of a soil (distilled water only) caused numbers to decline by 2.5 log_10_ cfu over the same period.

PHMB-treated gloves effectively reduced the numbers of *S. pyogenes*, CREC and MRSA by 4.50 log_10_ to below the detection limit (2 cfu) and *K. pneumoniae* by 2.50 log_10_ within 10 min of contact time when a soil-challenge was absent (distilled water only).

When the organic challenge was increased to moderate soiling (0.5% BSA) PHMB-treated gloves achieved 2.5 log_10_ reductions (<99.9%) in *S. pyogenes*, CREC, MRSA and *K. pneumoniae* contamination after 15 min exposure. When a heavy soil was present (blood), reductions were only <1 log_10_.

### Transfer from glove-tip to surface

Finger-pads of PHMB-treated and untreated (control) gloves were inoculated with approximately 2.5 × 10^4^- 4.8 × 10^4^ (i.e. 4.41 log_10_–4.68 log_10_) cfu bacteria prior to touching the recipient surface. Regardless of the soil type, similar numbers of bacteria could be transferred from an untreated or PHMB-treated glove to an uncontaminated keyboard key when the inoculum was wet (Table 2).

**Table 2:** Transfer of bacteria from recently contaminated fingertips (wet inoculum) and old contamination (dry inoculum) to a donor surface (keyboard key) and when in the presence of different soiling conditions; *n* = 10

	Median Log_10_ CFUs transferred (Inter-Quartile Range)					
Organism	Un-treated (control) glove			PHMB-treated glove		
Wet Inoculum	No soiling (DW)	Moderate soiling (0.5% BSA)	Heavy soiling (Blood)	No soiling (DW)	Moderate soiling (0.5% BSA)	Heavy soiling (Blood)
MRSA	4.20 (4.15–4.21)	4.08 (4.06–4.11)	4.16 (4.06–4.20)	4.06 (3.96–4.07)	4.07 (4.05–4.08)	3.99 (3.95–4.09)
Carbapenem-resistant E.coli	4.12 (4.09–4.15)	4.17 (4.12–4.11)	4.13 (4.12–4.23)	4.08 (4.08–4.13)	4.10 (4.08–4.13)	4.19 (4.16–4.26)
K. pneumoniae	4.39 (4.36–4.40)	4.29 (4.25–4.45)	4.20 (4.19–4.34)	4.39 (4.25–4.40)	4.28 (4.27–4.31)	4.35 (4.29–4.39)
S. pyogenes	4.54 (4.47–4.59)	4.65 (4.60–4.66)	4.75 (4.73–4.780	4.59 (4.25–4.40)	4.54 (4.41–4.57)	4.52 (4.45–4.56)
Dry Inoculum	No soiling (DW)	Moderate soiling (0.5% BSA)	Heavy soiling (Blood)	No soiling (DW)	Moderate soiling (0.5% BSA)	Heavy soiling (Blood)
MRSA	4.09 (4.07–4.20)	4.21 (4.20–4.23)	4.21 (4.17–4.23)	<0.301	<0.301	1.68 (1.64–1.72)
Carbapenem-resistant E.coli	3.74 (3.69–3.80)	4.12 (4.10–4.16)	4.19 (4.15–4.27)	<0.301	<0.301	2.12 (2.05–2.40)
K. pneumoniae	4.36 (4.33–4.39)	4.34 (4.29–4.40)	4.43 (4.39–4.48)	<0.301	<0.301	1.38 (1.38–1.60)
S. pyogenes	4.29 (4.26–4.34)	4.59 (4.57–4.61)	4.66 (4.58–4.69)	<0.301	<0.301	2.77 (2.70–2.82)

Note: Numbers below the detection limit (2 CFU) are expressed as <0.301 Log_10_ numbers

When the contaminating inoculum was dry, untreated-gloves were able to transfer the same numbers of bacteria to a keyboard surface as when the contamination was wet (under all soil conditions; Table 2). However, no contamination was detected (detection limit: 2 cfu) on keyboard keys that were touched with a dried inoculum on PHMB-treated gloves (distilled water and 0.5% BSA soil). When dry contaminated PHMB-treated gloves were soiled with blood, between 1.5–2.5 log_10_ bacteria could be transferred to the recipient surface.

### Transfer from contaminated surfaces to gloves or other surface

Keyboard keys were contaminated with approximately 3.3 × 10^4^- 8.2 × 10^4^ (i.e. 4.52 log_10_–4.91 log_10_) cfu bacteria (*S. pyogenes*, CREC, MRSA and *K. pneumoniae*) suspended in 0.5% BSA, blood or no soil (distilled water).

When recently contaminated (i.e. wet) keyboard keys were touched with the fingertip of PHMB-treated and untreated gloves, approximately 3–4 log_10_ bacteria were transferred to these finger tips (Table 3). Where the inoculum on the keyboard surface was dry (15 min incubation at room temperature) fewer numbers of bacteria could be transferred to the fingerpad (2.5–3.3 log_10_ bacteria) of both test and control gloves.

**Table 3:** Transfer from a donor surface (keyboard key) contaminated recently with bacteria (wet inoculum) or from old contamination (dry inoculum) to the fingertip of a gloved hand and when in the presence of different soiling conditions; *n* = 10

	Median Log_10_ CFUs transferred (Inter-Quartile Range)					
Organism	Un-treated (control) glove			PHMB-treated glove		
Wet Inoculum	No soiling (DW)	Moderate soiling (0.5% BSA)	Heavy soiling (Blood)	No soiling (DW)	Moderate soiling (0.5% BSA)	Heavy soiling (Blood)
MRSA	3.15 (3.04–3.28)	3.04 (3.00–3.46)	3.20 (3.08–3.34)	3.18 (3.04–3.28)	3.11 (3.09–3.20)	3.23 (3.20–3.32)
Carbapenem-resistant E.coli	3.34 (3.23–3.46)	3.18 (3.15–3.34)	3.30 (3.20–3.32)	3.00(2.95–3.08)	3.20 (2.70–3.23)	3.34 (3.30–3.49)
K. pneumoniae	4.03 (4.00–4.17)	4.12 (3.95–4.16))	4.01 (3.91–4.19)	3.34 (3.34–3.38)	3.41 (3.40–3.45)	3.89 (3.78–3.96)
S. pyogenes	3.93 (3.83–3.98)	3.93 (3.89–3.97)	3.96 (3.90–4.02)	3.94 (3.90–3.95)	3.95 (3.87–4.00)	3.99 (3.97–4.07)
Dry Inoculum	No soiling (DW)	Moderate soiling (0.5% BSA)	Heavy soiling (Blood)	No soiling (DW)	Moderate soiling (0.5% BSA)	Heavy soiling (Blood)
MRSA	2.78 (2.65–2.90)	2.78 (2.70–2.88)	3.13 (2.95–3.23)	2.73 (2.71–2.92)	2.80 (2.71–2.80)	3.35 (3.31–3.47)
Carbapenem-resistant E.coli	2.65 (2.65–2.74)	2.88 (2.70–2.88)	2.81 (2.78–2.88)	2.52 (2.52–2.65)	2.62 (2.43–2.68)	3.00 (2.92–3.28)
K. pneumoniae	3.13 (3.19–3.34)	3.28 (3.13–3.39)	3.53 (3.48–3.57)	2.65 (2.65–2.70)	2.65 (2.60–2.65)	2.48 (2.48–2.70)
S. pyogenes	3.27 (3.19–3.34)	3.30 (3.24–3.54)	3.44 (3.40–3.49)	3.13 (3.02–3.16)	3.34 (3.29–3.39)	3.36 (3.26–3.41)

Note: Numbers below the detection limit (2 CFU) are expressed as <0.301 Log_10_ numbers

### Transfer of bacteria from donor to recipient surfaces via gloves

Touching a wet donor (i.e. previously contaminated) keyboard key with the fingertip of either PHMB-treated or untreated gloves and then immediately touching an uncontaminated recipient keyboard key transferred approximately 2.4–3.6 log_10_ cfu bacteria (Table 3).

In the presence or absence of a moderate soiling (i.e. 0.5% BSA or DW respectively) on the donor surface, between 1.0–1.9 log_10_ cfu bacteria (S. *pyogenes*, CREC and *K. pneumoniae*) could be transferred with an untreated glove to the recipient surface when the contamination was dry (i.e. present on surface for 15 min).

Under the same conditions, between 0.3–0.6 log_10_ cfu bacteria were transferred to recipient surfaces using the PHMB-treated gloves. The numbers of MRSA transferred from donor to recipient surfaces were the same whether treated or untreated gloves were donned. No difference was observed in the numbers of bacteria transferred from donor to recipient surfaces using either treated or untreated gloves when blood soiling was present (*P* > 0.05).

## Discussion

The most frequent causes of healthcare-associated infections are *E. coli* (15.9%), *S. aureus* (12.3%) and *Klebsiella* spp. (8.7%) [1]. All of these may be spread on hands. Hand hygiene compliance varies widely depending on the staff category, clinical setting, and whether gloves are worn during patient care [7, 8].

Gloves are an essential part of personal protective equipment but not a substitute for hand hygiene. The use of medical examination gloves during patient-care is recommended as a “barrier” to infectious organisms in cases where contact with bodily fluids, mucous membranes or damaged skin is likely. However gloves should always be removed and hands decontaminated when moving away from the immediate patient-environment [9, 10]. The inappropriate use of gloves has been implicated in the cross transmission of clinically significant pathogens in the ICU setting [11]. In one study, it was shown only 5% of healthcare workers wearing gloves were likely to contaminate the skin of their hands after routine patient care compared to not wearing gloves (37%), demonstrating the benefits of a “barrier-protection” [12].

The various activities associated with patient care increase the likelihood of transmission of organisms from an infected patient to surfaces in their vicinity, especially in situations where there is repeated contact between staff, patient and environment. For example, during induction of anaesthesia the number of gloved-hand contact between the patient and environment is too frequent to allow hand-hygiene on every occasion.

In an effort to prevent the transmission of micro-organisms during procedures involving patient contact, double-gloving of the hands was implemented in an anaesthetic operating room. Removal of the outer glove of a double-layer significantly reduced the presence of contaminating organisms in the intraoperative environment, suggesting the rapid elimination of microorganisms on the surfaces of the hands may reduce the risk of cross-transmission from surfaces near patients. However, the implementation of double-layer gloving to complement hand hygiene may not be practical in emergency care situations. Consequently, the use of gloves impregnated with antimicrobial agents would be expected to reduce the risk of transmission from a patient to their environment and then to another patient [8].

PHMB is a broad spectrum antimicrobial agent supported under Directive 98/8/EC for use as a disinfectant. It is used as a preservative and as an antimicrobial agent in wet wipes, to prevent microbial contamination in wound irrigant or gel and sterile dressings, and to disinfect hard surfaces for food handling in institutions and hospitals. In a randomised controlled trial of impregnated dressings, it was superior to silver in reduction of bacterial load in chronic wounds [13]. However the EU Scientific Committee did not recommend its use in cosmetics as 0.3% preservative or spray-formulation [14].

In this study we showed PHMB-treated nitrile gloves can reduce *S. pyogenes*, CREC and MRSA contamination by ~4.5 log_10_ cfu (~99.99% reduction) to below the detection limit (2 cfu) within 10 min of contact time. There is an approximate 2.5 log_10_ cfu reduction of *K. pneumoniae*. This demonstrates a potential for PHMB-treated gloves to reduce the risk of contaminating nearby surfaces. Both PHMB-treated and untreated glove types could transfer high numbers of contaminating bacteria to uncontaminated surfaces if still wet. However, PHMB-treated gloves that were donned for 15 min (i.e. when the contaminating inoculum was visibly dry) did not transfer bacteria to uncontaminated surfaces in the absence of soil or when only moderate soiling (0.5% BSA) was present.

The concentration of contaminating organisms carried in an inoculum may influence the degree of cross-contamination. Hands or gloves contaminated with a small inoculum concentration may result in greater percentage transfer to an un-contaminated surface than a large inoculum [15]. Therefore, reductions in bacterial numbers to low numbers on a glove using antimicrobial PHMB may not directly correlate to a relative risk-reduction in potential cross-contamination of these organisms. Nonetheless, the efficacies demonstrated in our study show numbers may be reduced to below the detection limit (2 cfu) in short contact times (i.e. 10 min). The risk of spread could be further minimised if gloves are removed and changed between frequently and between activities.

A criticism of the study is that glove fingertip pads were homogenized to remove bacteria instead of using a swabbing technique. Homogenisation of the excised fingerpad of the glove allows the capture of bacterial cells from the microscopic contours of the glove material that may otherwise be inaccessible if using a swab. The antimicrobial PHMB additive was impregnated uniformly throughout the treated-glove material during manufacture and not applied superficially, thus minimising variation between structure and microscopic surface with the untreated (control) glove. More bacteria may be deposited from a glove than from a bare hand [16]. While swabbing will indicate the potential transfer of superficial contaminating cells homogenisation will permit the isolation of cells transferred in successive contacts to uncontaminated surfaces.

The application of antimicrobial PHMB in medical examination gloves has been evaluated recently [17]. However, in that assessment, the contaminating numbers were low (up to 10^2^ cfu) and did not address the potential risks of cross-transfer to uncontaminated surfaces. In another study the antimicrobial efficacy of PHMB impregnated gloves was enhanced with the application of pressure (75 g weight for 1 min) to increase contact with the inoculum and therefore facilitating antimicrobial activity [18].

The antimicrobial potential of gloves impregnated with a variety of antimicrobial agents other than PHMB have been explored, though the design and test-criteria for the selection and development of antimicrobial gloves vary depending on the end-use and requirement pre-determined by the investigator.

Gentian violet and chlorhexidine impregnated into PVA gloves demonstrated rapid bactericidal activity against several clinically relevant pathogens (MRSA, vancomycin-resistant enterocci, multidrug-resistant *Pseudomonas aeruginosa*, and a carbapenemase producing *Klebsiella pneumoniae*) when assessed following the criteria described within the JIS Z 2801 standard for the determination of antimicrobial non-porous surfaces [19]. However, this test requires the application of glass cover-slips to increase surface contact and does not incorporate an interfering-substance/soil challenge, therefore permitting “enhanced” in-vitro antimicrobial activity that may not be achievable under realistic conditions. In the current study, antimicrobial activity was assessed without the application of enhanced pressure or cover slips to maximise contact. The presence of heavy soils, such as blood, limited bacterial reduction on PHMB-treated gloves to <1 log_10_ cfu. Nonetheless, the transfer of bacteria from PHMB-treated glove was significantly less compared with untreated gloves regardless of whether the contamination was recent or up to 15 min old (*P* < 0.05).

During use, micro-perforation of the medical examination glove is common [20, 21]. Loss of structural integrity may result in the bi-directional migration of contaminating organisms between the hands of the HCW, the patient and proximal surfaces in the clinical environment. In one study, chlorhexidine gluconate in a matrix on the inside of gloves was used to protect the staff member from accidental puncture contamination with body fluids [22]. In another study, coating antimicrobial chlorhexidine digluconate onto the inner surfaces of a glove demonstrated antimicrobial efficacy against *S. aureus* and *K. pneumoniae* for up to two-hours of continued use in a surgical setting [23].

A study by Reitzel, et al. showed rapid bactericidal activity for gloves impregnated with brilliant green and chlorhexidine but the organic solvents required in the process compromised glove integrity [19]. Compatibility of the glove material with the antimicrobial agent and the intended use should therefore be addressed prior, as disintegration of the glove material may expose the wearer to potentially pathogenic organisms. In the current study, the inner surfaces of the treated gloves used in the current study were not assessed for antimicrobial activity. However, the PHMB agent used was distributed throughout the inner and outer surfaces of the glove. This property indicates PHMB-impregnated gloves may reduce the risk of bacterial transfer upon puncture of the glove material. Although not assessed, the nitrile examination gloves evaluated in our study were designed to be compatible with the PHMB antimicrobial additive and did not compromise the physical integrity of the material during testing.

In conclusion, the use of antimicrobial nitrile gloves has a potential benefit in the clinical environment in reducing accidental environmental contamination. Soiling (e.g. blood) significantly reduces the efficacy of such materials so the gloves will have less effect on transfer of organisms between different sites on the patient. The test criteria for the evaluations of antimicrobial activity should be based upon realistic measures and relevance to the application and setting.

The use of medical examination gloves treated with antimicrobial agents (e.g. PHMB) should be utilised in conjunction with appropriate hand hygiene practices and gloves changed frequently between activities and after prolonged use.
